# Protective Effects of Corni Fructus against Advanced Glycation Endproducts and Radical Scavenging

**DOI:** 10.1155/2012/418953

**Published:** 2012-05-09

**Authors:** Chan Hum Park, Takashi Tanaka, Hyun Young Kim, Jong Cheol Park, Takako Yokozawa

**Affiliations:** ^1^Institute of Natural Medicine, University of Toyama, Toyama 930-0194, Japan; ^2^Graduate School of Biomedical Science, Nagasaki University, Nagasaki 852-8521, Japan; ^3^Department of Food Science, Gyeongnam National University of Science and Technology, Gyeongnam 660-758, Republic of Korea; ^4^Department of Oriental Medicine Resources and Research Institute of Korean Oriental Medicines, Sunchon National University, Jeonnam 540-742, Republic of Korea

## Abstract

We investigated the inhibition of advanced glycation endproduct (AGE) activity using the fluorescence characteristics of fractions and compounds from Corni Fructus. Corni Fructus extract and its iridoid glycoside components showed low inhibitory activities as well as the AGE inhibitor aminoguanidine. However, a low molecular weight polyphenol, 7-*O*-galloyl-D-sedoheptulose, and an antioxidant, trolox, showed high inhibitory activities compared with aminoguanidine under reactive conditions. The AGE-inhibiting activity of polyphenolic fractions of Corni Fructus ranged from a level comparable to Corni Fructus extract to the higher level of 7-*O*-galloyl-D-sedoheptulose. As well as the results of AGE-inhibiting activity, Corni Fructus extract and iridoid components showed low or no 1,1-diphenyl-2-pycrylhydrazyl (DPPH) radical-scavenging activities, whereas 7-*O*-galloyl-D-sedoheptulose showed a level comparable to trolox. Polyphenolic fractions of Corni Fructus quenched DPPH radicals in a concentration-dependent manner. Some fractions exerted a higher DPPH radical-scavenging activity compared with trolox and 7-*O*-galloyl-D-sedoheptulose. The DPPH radical-scavenging activity was significantly correlated with the AGE-inhibiting activity. These results suggest that polyphenolic fractions of Corni Fructus inhibited AGE formation by antioxidant activity including free radical scavenging. The strong DPPH radical-scavenging and AGE-inhibiting fractions included ellagitannins and polymeric proanthocyanidins.

## 1. Introduction

The advanced glycation endproduct (AGE), which is the nonenzymatic modification of proteins by reducing sugars, plays an important role in the development of chronic diabetic complications [1, 2] and aging [2]. Therefore, the inhibition of AGE formation may be a promising target for therapeutic intervention in these AGE-related disorders.

 AGE inhibitors may act through various mechanisms at different steps of AGE formation and AGE-mediated damage, such as the trapping of reactive dicarbonyl species, antioxidant activity by transition metal chelation, other antioxidant activity including free radical scavenging, AGE cross-link cleavage, AGE receptor blocking, AGE signaling blocking, glycemia reduction by antidiabetic therapy, aldose reductase inhibition, and shunting of trioses-P towards the pentose-P pathway by transketolase activation [3]. Although some AGE inhibitors such as aminoguanidine, OPB-9195, LR compounds (LR-90, LR-9, and LR-74), and TM2002 have been developed, no agent is used for diabetic complications in Japan at the present time. One of the reasons for the difficulty in the development of AGE inhibitors is the side effects. In the case of aminoguanidine, a phase III clinical trial was started [4]; however, the study was discontinued due to safety concerns and an apparent lack of efficacy [3].

 From this standpoint, traditional medicines and their components have the advantage that they act against the development of AGE without side effects. We have investigated the therapeutic potential of traditional medicines against diabetic nephropathy associated with oxidative stress and AGE formation. We previously reported the effects of 12 oriental medicines and their galenicals on the production of AGE *in vitro*, and polyphenol-containing galenicals exerted high-level inhibitory activity against AGE production, assumed to be due to the elimination of free radicals [5]. Since polyphenols exerted marked radical-scavenging activity [6, 7], this suggested the involvement of antioxidant activity, including free radical scavenging, in AGE inhibition by polyphenols.

 In subsequent studies, the Chinese prescription Hachimi-jio-gan, which is prescribed for diabetes and diabetic nephropathy, exhibited potential therapeutic effects against metabolic disorders, especially AGE formation [8]. In addition, Corni Fructus, which is an ingredient of Hachimi-jio-gan and has been used as a functional food, showed beneficial effects on renal metabolic abnormalities including AGE formation in streptozotocin-induced diabetic rats as well as those of Hachimi-jio-gan [9, 10]. Corni Fructus is known to contain iridoid glycosides, such as morroniside and loganin, and polyphenolic compounds. Morroniside can prevent diabetic renal damage via inhibiting AGE-receptor for AGE (RAGE) binding rather than AGE formation as well as oxidative stress in type 1 diabetic rats [11]. Moreover, loganin can prevent diabetic hepatic damage via inhibiting AGE and RAGE through improving hyperglycemia and dyslipidemia in type 2 diabetic mice [12]. On the other hand, polyphenolic compounds, such as 7-*O*-galloyl-d-sedoheptulose, proanthocyanidins, and ellagitannins, were also isolated [13–15]. Recently, we reported that 7-*O*-galloyl-d-sedoheptulose shows a lipid-lowering effect on the liver in hypertriglyceridemic rats, and a protective effect against oxidative stress [16], AGE and RAGE in the liver or kidney of type 1 and 2 diabetic animal models [17, 18]. The polyphenolic fraction containing polymeric proanthocyanidins has the potential to suppress postprandial hyperglycemia, which facilitates AGE formation [19]. These results indicate that several constituents of Corni Fructus inhibit AGE formation by different mechanisms. AGE formation is increased under conditions of oxidative stress [20]. Glycation and oxidative stress are closely linked and are often referred to as “glycoxidation” processes. All glycation steps generate oxygen-free radicals [21]. In addition, phenolic-rich plants have tended to have antioxidant and antiglycation activity. Therefore, to elucidate the effects of fractions and compounds from Corni Fructus against AGE formation, we investigated the AGE-inhibiting activity using fluorescence characteristics, and also radical-scavenging activity using the stable 1,1-diphenyl-2-pycrylhydrazyl (DPPH) radical.

## 2. Materials and Methods

### 2.1. General

Column chromatography was performed using Diaion HP20SS (Mitsubishi Chemical, Tokyo, Japan), Sephadex LH-20 (25–100 *μ*m, GE Healthcare Bio-Sciences AB, Uppsala, Sweden) and Chromatorex ODS-MT (100–200 mesh; Fuji Silysia Chemical, Kasugai, Japan) columns. Thin-layer chromatography (TLC) was performed on 0.2-mm-thick precoated Kieselgel 60 F_254_ plates (Merck, Darmstadt, Germany) using toluene-ethyl formate-formic acid (1 : 7 : 1, v/v) or chloroform-MeOH-H_2_O (14 : 6 : 1, v/v). Spots were detected by UV illumination, sprayed with 2% methanolic FeCl_3_ or 10% sulfuric acid reagent, and then heated. Analytical reverse-phase HPLC was performed on a Cosmosil 5C_18_-AR II column (Nacalai Tesque Inc., Kyoto, Japan; 4.6 mm i.d. × 250 mm) using an elution gradient of 4–30% (39 min) and 30–75% (15 min) CH_3_CN in 50 mM H_3_PO_4_ (flow rate: 0.8 mL/min; detection using the JASCO photodiode array detector MD-910). Gel permeation column chromatography was performed with a TSK-gel *α*3000 column (300 × 7.8 mm i.d.) at 40°C with dimethylformamide containing 10 mM LiCl as the elution solvent at a flow rate of 0.5 mL/min. The peaks were detected by monitoring UV absorption at 254 nm. Polystyrene standards (molecular weights of 4,000, 25,000, 50,000, and 170,000), and toluene (molecular weight of 92) were used as standards. The molecular weights were recorded and calculated employing integrator 807-IT (JASCO Corporation). The ^1^H- and ^13^C-NMR spectra were recorded in a mixture of acetone-*d*_6_ and D_2_O (19 : 1, v/v) at 27°C with a JEOL JNM-AL400 spectrometer operating at 400 MHz for ^1^H and 100 MHz for ^13^C (JEOL Ltd., Tokyo, Japan). Aminoguanidine and trolox were purchased from Sigma Chemical Co. (St. Louis, MO, USA). DPPH was purchased from Wako Pure Chemical Industries, Ltd. (Osaka, Japan).

### 2.2. Plant Material

Corni Fructus (*Cornus officinalis* Sieb. et Zucc.) was purchased from Uchida Wakanyaku Ltd., Tokyo, Japan.

### 2.3. Extraction, Fractionation, and Isolation

**Figure 1:**
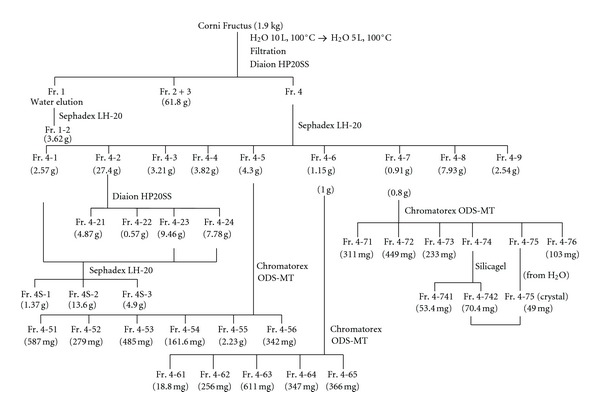
Fractionation of Corni Fructus extract.

**Figure 2:**
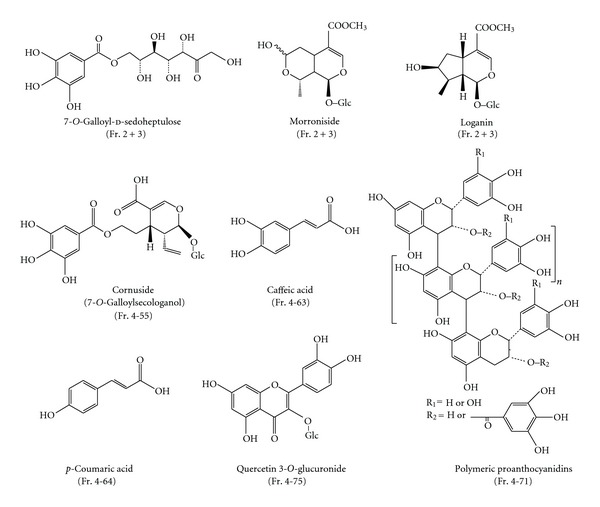
Chemical structure of compounds isolated from Corni Fructus extract. ( ) has been presented the fraction which was isolated.

Corni Fructus (1.9 kg) was crushed using a Warring blender and then extracted with boiling water (10 L) for 10 min. After filtration, the plant debris was extracted again with 5 L of boiling water. The filtrates were combined and directly applied to a Diaion HP20SS (8 × 42 cm) column. The column was eluted with H_2_O containing increasing proportions of MeOH (0–100%, 20% stepwise, each at 1 L) and finally with 60% acetone. The eluate was monitored by TLC and HPLC and separated into 4 fractions (Figure 1). Fractions 1, 2, and 3 mainly contained sugars, 7-*O*-galloyl-d-sedoheptulose, and iridoid glycosides, respectively, and were not examined further because of the low-level activities of these compounds. Fraction 4 contained polyphenols and iridoid glycosides. The major polyphenols were polymeric proanthocyanidins, which can be detected at the origin on TLC analysis and as a broad hump on the HPLC baseline. Fraction 4 (59.0 g) was subjected to Sephadex LH-20 column chromatography (10 × 24 cm) with H_2_O containing increasing proportions of MeOH (0–100%, 20% stepwise, each at 500 mL) and finally with 60% acetone to yield 9 fractions. Fractions 4-1 and 4-2 were separately applied to Diaion HP20SS and Sephadex LH-20, and the fractions containing the same compounds were combined to give the fractions 4S-1, 4S-2, and 4S-3. The proanthocyanidins were dispersed in the fractions (4S-1, 4S-3, 4-4, 4-62, 4-71, and 4-8), and the total yield was about 1.2%. The fractions containing proanthocyanidins were characterized by thiol degradation [22], and the products were identified as epigallocatechin-4-hydroxyethylthioether (23.5 min), epigallocatechin 3-*O*-gallate-4-hydroxyethylthioether (28.3 min), epicatechin-4-hydroxyethylthioether (29.0 min), and epicatechin 3-*O*-gallate-4-hydroxyethylthioether (35.2 min) [18]. Fraction 4-5 was separated by Chromatorex ODS-MT column chromatography to yield 6 fractions. The major fraction, fraction 4-55, was identified as cornuside (7-*O*-galloylsecologanol) [14] by ^1^H- and ^13^C-NMR comparison. Fraction 4-6 was subjected to Chromatorex ODS-MT column chromatography to yield 5 fractions. The major fractions, fractions 4-63 and 4-64, were identified as caffeic and *p*-coumaric acids, respectively. Similar column chromatography of fraction 4-7 yielded 6 fractions. Fraction 4-71 was composed of polymeric proanthocyanidins. Fraction 4-75 was identified as quercetin 3-*O*-glucuronide by ^1^H- and ^13^C-NMR comparison. Fraction 4-8 mainly contained polymeric proanthocyanidins along with a small amount of ellagitannins. Fraction 4-9 gave a positive result in the presence of NaNO_2_-AcOH reagent and yielded ellagic acid and gallic acid on acid hydrolysis (2 mg/mL in 2% HCl, 100°C, 2 h, identified by HPLC comparison), indicating that this fraction was mainly composed of ellagitannins. The chemical structure isolated from each fraction is shown in Figure 2.

### 2.4. Determination of AGE

 According to the method of Vinson and Howard [23], bovine serum albumin (10 mg/mL) in 50 mM phosphate-buffer (pH 7.4) with 0.02% sodium azide to prevent bacterial growth was added to glucose (25 mM) and fructose (25 mM). This reaction mixture was mixed with different concentrations (12.5, 25, 50, 100 *μ*g/mL) of test sample or aminoguanidine and trolox. After incubation at 37°C for 2 weeks, the fluorescent reaction products were assayed on a spectrofluorometric detector (Shimadzu RF-550, Kyoto, Japan) with the excitation at 350 nm and emission at 450 nm. All incubations were done in quadruplicate. The data are expressed in terms of the IC_50_ value (concentration in *μ*g/mL required to inhibit AGE formation by 50%) calculated from the log-dose inhibition curve.

### 2.5. Determination of DPPH Radicals

In microwells, 100 *μ*L of an aqueous solution of the sample (control: 100 *μ*L of distilled water) was added to an ethanolic solution of DPPH (60 *μ*M) according to the method of Hatano et al. [6]. Seven concentrations were prepared for each sample. After mixing gently and leaving to stand for 30 min at room temperature, the optical density was determined using a Microplate Reader, model 3550-UV (Bio-Rad). The antioxidant activity of each sample was expressed in terms of the IC_50_ (micromolar concentration required to inhibit DPPH radical formation by 50%), calculated from the log-dose inhibition curve. 

### 2.6. Statistical Analysis

 Data are expressed as the mean ± SEM. Simple regression analysis was performed to investigate the correlation between DPPH radical-scavenging and AGE-inhibiting activities using the Microsoft Excel 2003 statistical package.

## 3. Results

### 3.1. AGE-Inhibiting Activity

**Figure 3:**
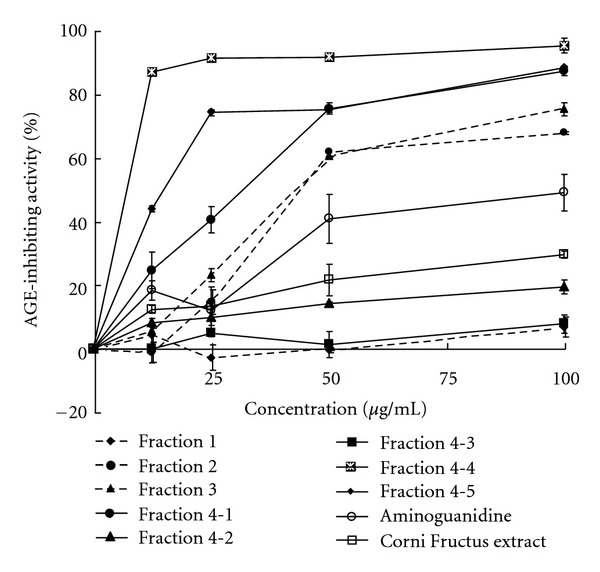
AGE-inhibiting activity of Corni Fructus fractions.

The AGE-inhibiting activities of 8 fractions of Corni Fructus showed wide variation. Fractions 4-4 and 4-5 exerted higher inhibition compared with aminoguanidine at all tested concentrations (Figure 3).

 Next, subfractions from fractions 4-5, 4-6, and 4-7, and fractions 4-8 and 4-9 were evaluated regarding their inhibitory activity. As shown in Table 1, most of the test samples inhibited AGE formation. Aminoguanidine showed low or no inhibitory activities against AGE formation under reactive conditions. Trolox, known as an antioxidant, showed more marked inhibition compared with aminoguanidine. Corni Fructus extract and iridoid glycoside components, morroniside and loganin, showed low inhibitory activity as well as aminoguanidine. On the other hand, the inhibitory activity of 7-*O*-galloyl-d-sedoheptulose was higher than that of other references. Fractions of Corni Fructus extract ranged from a level comparable to Corni Fructus extract to the higher level of 7-*O*-galloyl-d-sedoheptulose. Fraction 4-9, containing ellagitannin, exerted the highest AGE-inhibiting activity of all samples, as shown in Table 1.

### 3.2. DPPH Radical-Scavenging Activity

As shown in Table 1, trolox showed DPPH radical-scavenging activity. Although Corni Fructus extract and iridoid components showed low or no DPPH radical-scavenging activity, 7-*O*-galloyl-d-sedoheptulose showed a level comparable to trolox under reactive conditions. Polyphenolic fractions of Corni Fructus were quenched with DPPH radicals, and some fractions exerted higher DPPH radical-scavenging activity compared with trolox and 7-*O*-galloyl-d-sedoheptulose. Fraction 4-9, containing ellagitannin, exerted the highest DPPH radical-scavenging activity of all samples.

### 3.3. Correlation between AGE-Inhibiting and DPPH Radical-Scavenging Activities

**Figure 4:**
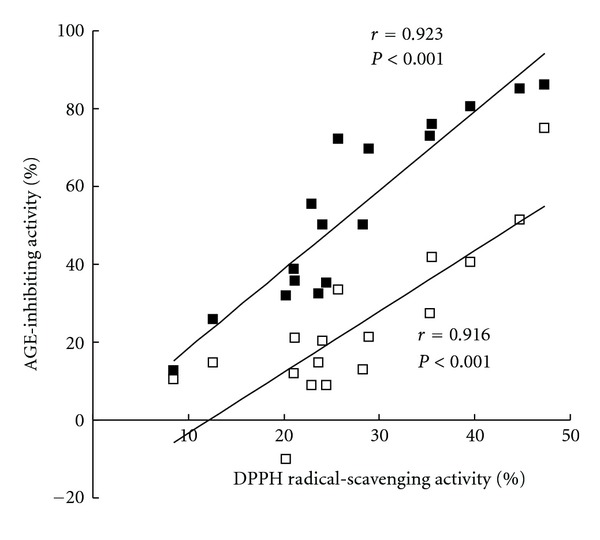
Correlation between AGE-inhibiting and DPPH radical-scavenging activities of Corni Fructus polyphenolic fractions. The correlation between AGE-inhibiting activity at a concentration of 12.5 *μ*g/mL and DPPH radical-scavenging activity at a concentration of 1 *μ*g/mL (■), and AGE-inhibiting activity at a concentration of 25 *μ*g/mL and DPPH radical-scavenging activity at a concentration of 2 *μ*g/mL (□).

The correlation between AGE-inhibiting activity at a concentration of 12.5 *μ*g/mL and DPPH radical-scavenging activity at a concentration of 1 *μ*g/mL is shown in Figure 4 (■), indicating a significant positive correlation. Also, a similar positive correlation between AGE-inhibiting activity at a concentration of 25 *μ*g/mL and DPPH radical-scavenging activity at a concentration of 2 *μ*g/mL was observed, as shown in Figure 4 (□).

## 4. Discussion

Polyphenols are natural products that demonstrate antioxidative activity in the form of anti-carcinogenetic, anti-cardiovascular disease, and antimelanogenic activities. In this paper, we evaluated AGE-inhibiting and DPPH radical-scavenging activities of fractions of Corni Fructus. The results showed that fractions containing ellagitannins or polymeric proanthocyanidins exerted marked DPPH radical-scavenging and AGE-inhibiting activities. The strong inhibitory activities of fraction 4-4 (Figure 3) were also attributable to the polymeric proanthocyanidins, which are characterized by thiol degradation [22]. In the fractionation, polymeric proanthocyanidins were dispersed into some fractions obtained by Sephadex LH-20 column chromatography. The reason is not clear; however, proanthocyanidin-pectin complexation may be affected during separation because Corni Fructus is from dried fruit and contains a large amount of pectin [24].

 The DPPH radical is stable and has been used as a tool for radical-scavenging assays. In this study, some fractions contained polymeric proanthocyanidins, and ellagitannins exerted high-level DPPH radical-scavenging activity compared with 7-*O*-galloyl-d-sedoheptulose. The free radical-scavenging effects of polyphenols and related polyphenols having an *o*-trihydroxyl (pyrogallol) structure (galloyl, hexahydroxydiphenoyl groups in hydrolyzable tannins, a galloyl group in acylated proanthocyanidins, and the B-ring of some flavan-3-ols) were stronger than the effects of unacylated proanthocyanidins [6, 7]. The scavenging activity was elevated with an increase of the molecular weight [6]. The differences in DPPH radical-scavenging activity among the fractions may be due to the molecular weight and rate of galloylation. The antioxidant activity of hydrolyzable tannins and tannic acid is mainly due to iron chelation rather than hydroxyl radical scavenging [25]. In this study, fraction 4-9 exhibited the highest DPPH radical-scavenging and highest AGE-inhibiting activities. This fraction included ellagitannins and polymeric proanthocyanidins. Ellagitannins were detected almost exclusively in this fraction and characterized by hydrolysis yielding ellagic acid; however, the structures could not be identified due to difficulty of purification and complexity of the composition. The highest activity of fraction 4-9 could be due to their pyrogallol structure and transition metal chelation. Further chemical investigation of the ellagitannin fraction is now in progress.

 In this study, trolox exerted high-level activity against both DPPH radical scavenging and AGE inhibition. Trolox, which is a water soluble analogue of *α*-tocopherol, has been proven to be an antioxidant under a wide range of conditions and test systems [26]. This result suggests that trolox inhibited AGE formation by antioxidant activity including free radical scavenging. The relationship between the DPPH radical-scavenging and AGE-inhibiting activities of polyphenolic fractions of Corni Fructus showed the same tendency as the results for trolox. This suggested that polyphenolic fractions of Corni Fructus also inhibited AGE formation by antioxidant activity including free radical scavenging.

 Both the DPPH radical-scavenging and AGE-inhibiting activities of trolox were higher than those of aminoguanidine. The mechanism of aminoguanidine, which is one of the AGE inhibitors, has been shown to involve the trapping of reactive dicarbonyl species [27], antioxidant activity by transition metal chelation [28], and other antioxidant activity including hydroxyl radical scavenging [28, 29]. Therefore, potent antioxidant activity is expected to play an important role in AGE inhibition.

 Biological or physiological studies of polyphenolic constituents of Corni Fructus are limited. In our previous studies, we reported the hepato- or reno-protective role of a low molecular weight polyphenol, 7-*O*-galloyl-d-sedoheptulose, on diabetes associated with AGE and oxidative stress in streptozotocin-induced type 1 diabetic rats [17] and type 2 diabetic *db/db* mice [18]. The fraction containing polymeric proanthocyanidins showed *α*-glucosidase-inhibiting activity and suppressed plasma glucose levels after sucrose loading [19]. In this study, marked AGE-inhibiting and DPPH radical-scavenging activities were found in several fractions containing polymeric proanthocyanidins. It is interesting that polymeric proanthocyanidins show not only a high AGE-inhibiting activity, but also DPPH radical-scavenging activity, and the limited fractions exhibit high *α*-glucosidase-inhibiting activity. This suggests that, in addition to 7-*O*-galloyl-d-sedoheptulose, another active component may be present in these polyphenolic fractions.

## 5. Conclusions

Some polyphenolic fractions of Corni Fructus exerted both AGE-inhibiting and DPPH radical-scavenging activities *in vitro*, suggesting the possibility of inhibiting AGE formation *in vivo*.

## Figures and Tables

**Table 1:** AGE-inhibiting and DPPH radical-scavenging activities of the fractions from Corni Fructus extract.

Sample	Major constituents	AGE-inhibiting activity (%)		DPPH radical-scavenging activity (%)	
		12.5 *μ*g/mL	25 *μ*g/mL	1 *μ*g/mL	2 *μ*g/mL
Fr. 4-74	Mix^a^	10.3 ± 1.0	12.6 ± 8.2	8.4 ± 0.5	14.4 ± 0.8
Fr. 4-56	Mix^a^	14.7 ± 0.6	25.7 ± 2.8	12.6 ± 0.6	15.7 ± 1.1
Fr. 4-65	Mix^a^	−10.1 ± 9.0	31.9 ± 1.8	20.3 ± 0.3	41.0 ± 1.1
Fr. 4-53	Mix^a^ + polymeric proanthocyanidins	11.9 ± 6.0	38.6 ± 1.3	21.1 ± 0.6	38.4 ± 0.3
Fr. 4-54	Mix^a^ + polymeric proanthocyanidins	20.9 ± 6.0	35.7 ± 0.9	21.2 ± 0.9	41.7 ± 0.9
Fr. 4-75	Quercetin glucuronide	8.9 ± 4.0	55.3 ± 5.2	22.9 ± 1.3	48.7 ± 0.6
Fr. 4-64	*p*-Coumaric acid	14.6 ± 10.6	32.4 ± 7.5	23.6 ± 1.6	39.5 ± 1.3
Fr. 4-52	Mix^a^	20.3 ± 1.7	50.0 ± 1.8	24.1 ± 0.6	37.2 ± 0.4
Fr. 4-51	Mix^a^ + polymeric proanthocyanidins	8.8 ± 3.8	35.2 ± 5.5	24.5 ± 1.5	39.0 ± 1.2
Fr. 4-76	Mix^a^	33.3 ± 5.8	72.3 ± 1.8	25.7 ± 1.1	48.0 ± 0.9
Fr. 4-55	Cornuside	13.0 ± 2.4	50.2 ± 1.0	28.3 ± 0.5	51.1 ± 0.3
Fr. 4-63	Caffeic acid + polymeric proanthocyanidins	21.2 ± 4.7	69.6 ± 0.8	28.9 ± 0.3	67.1 ± 0.1
Fr. 4-62	Mix^a^ + polymeric proanthocyanidins	27.3 ± 6.1	73.0 ± 0.7	35.3 ± 0.3	63.7 ± 0.6
Fr. 4-71	Polymeric proanthocyanidins	41.8 ± 2.9	76.1 ± 1.6	35.6 ± 1.3	64.1 ± 2.3
Fr. 4-72	Mix^a^ + polymeric proanthocyanidins	40.5 ± 2.6	80.5 ± 1.8	39.6 ± 0.9	68.2 ± 0.5
Fr. 4-8	Polymeric proanthocyanidins	51.3 ± 1.4	85.1 ± 0.3	44.7 ± 0.8	66.1 ± 1.2
Fr. 4-9	Ellagitannins + polymeric proanthocyanidins	75.0 ± 1.8	86.0 ± 0.5	47.3 ± 0.4	70.8 ± 1.8

Corni Fructus extract		9.2 ± 5.2	12.8 ± 0.5	2.8 ± 1.1	5.9 ± 1.4
Morroniside		5.9 ± 2.1	−0.2 ± 3.1	3.3 ± 0.3	2.1 ± 1.1
Loganin		4.0 ± 2.1	−0.6 ± 2.4	1.5 ± 2.2	−0.9 ± 1.6
7-*O*-Galloyl-d-sedoheptulose		48.2 ± 3.3	61.9 ± 3.7	36.5 ± 0.7	59.6 ± 0.7
Trolox		23.2 ± 2.7	34.1 ± 2.1	33.8 ± 0.5	60.7 ± 1.0
Aminoguanidine		−0.2 ± 1.5	8.6 ± 3.0	5.6 ± 2.1	0.8 ± 3.6

^a^Mixture of low molecular weight compounds.
